# Refractory Pigmentation Associated with Laugier-Hunziker Syndrome following Er:YAG Laser Treatment

**DOI:** 10.1155/2013/561040

**Published:** 2013-12-03

**Authors:** Sertan Ergun, Alp Saruhanoğlu, Dante-Antonio Migliari, Ilay Maden, Hakkı Tanyeri

**Affiliations:** ^1^Department of Oral Medicine and Surgery, Faculty of Dentistry, Istanbul University, 34093 Istanbul, Turkey; ^2^Department of Oral Diagnosis, School of Dentistry, University of Sao Paulo, 05508-900 Sao Paulo, SP, Brazil; ^3^Department of Periodontology, Istanbul Faculty of Medicine, Istanbul University, 34093 Istanbul, Turkey

## Abstract

The present report describes a case of Laugier-Hunziker syndrome (LHS), a rare benign condition. A patient with LHS develops acquired melanotic pigmentation of the lips and buccal mucosa, often with pigmentation of the nails occurring. No systemic symptoms are associated with this syndrome. Normally, no treatment is required for this condition, unless for aesthetic reason, mainly due to pigmentation on the lip mucosa. We present a case of LHS, 37-year-old female, whose pigmentations on her lip and in the oral cavity were treated with an Er:YAG laser. At the postoperative 12th month followup, the lesions recurred. The effects of any surgical attempt to treat pigmentations associated with LHS were discussed.

## 1. Introduction

The Laugier-Hunziker syndrome (LHS) is a rare benign condition, characterized by acquired pigmentation of the nails and melanotic pigmentation of the parts of the oral cavity such as lips, buccal, and palatal mucosa, [1]. Oral pigmentation is either focal or diffuse. Lip lesions and mucosa present as multiple, flat, smooth, discrete or confluent pigmented macules of variable size and color, ranging from grey to brown or blue-black [2].

Focal lesions may be more worrying, and require an examination of a biopsy specimen for an accurate diagnosis and, mainly, for excluding melanoma. Histopathological examination of oral pigmentation associated with LHS is not a diagnosis in itself; it only shows a significant increase in melanin or melanocytes in the basal layer. Diagnosis of LHS should be made on a clinical basis, by excluding other similar disorders such as Peutz-Jeghers syndrome and adrenal insufficiency [3].

It has been reported that very few patients actually receive treatment for LHS because it is a benign condition. We present a case of LHS treated with Er:YAG laser to remove the oral pigmentations which were cleared after four sessions but recurred twelve months later.

## 2. Case Report

**Figure 1:**
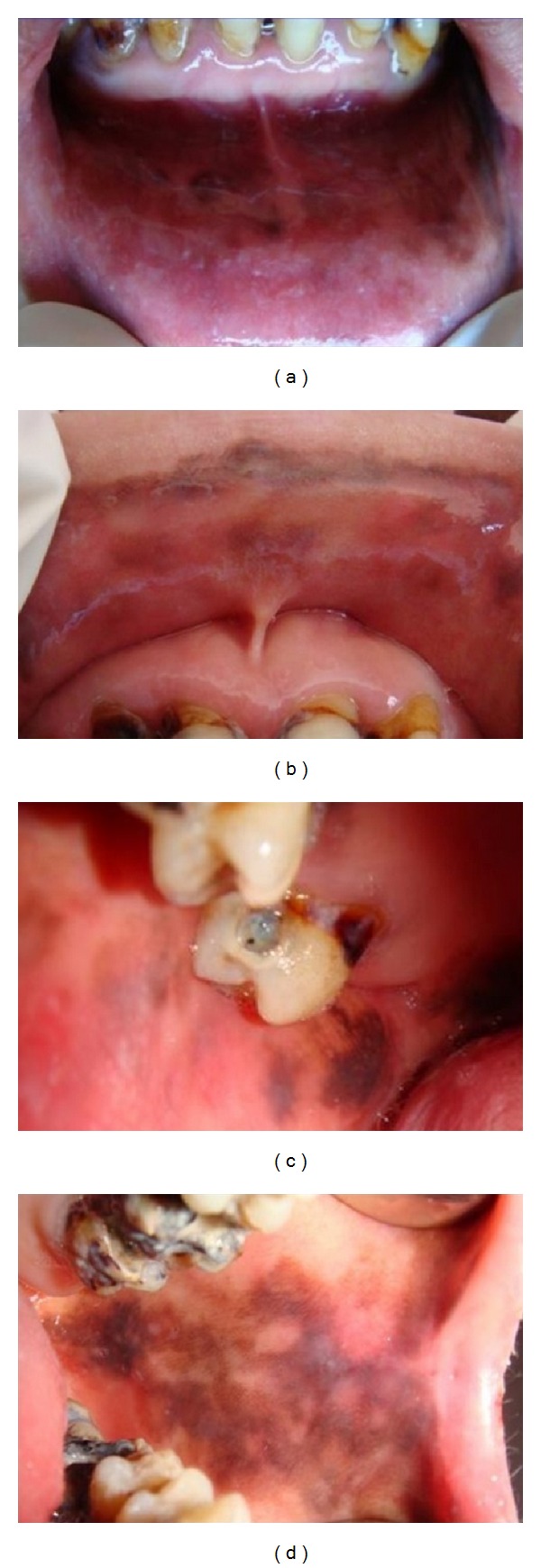
Multiple, painless brown-black pigmentation, on the buccal mucosa, lower lip, and posterior of the palate, bilaterally.

A 37-year-old Turkish woman was referred to the department of oral surgery because of pigmented areas in her mouth. She had noticed the pigmented lesions in her oral cavity 3 years ago and till the beginning of the treatment no major change in the oral cavity was observed by the patient. Oral examination revealed multiple, painless brown-black pigmentation, on the buccal mucosa, lower lip and posterior of the palate, bilaterally (Figure 1). No cutaneous or fingernail lesions were observed. She was systemically healthy and was not on any medication. She was neither a smoker nor a habitual drinker of alcohol. There was no family history of abnormal mucocutaneous pigmentation.

Laboratory investigation results, including a full blood count, hematinic levels, serum chemistry and inflammatory markers, were all within normal range. The patient underwent an upper gastrointestinal endoscopy, as well as a colonoscopy, which revealed no evidence of polyps. To rule out Addisons disease, the serum cortisol and adrenocorticotrophic hormone (ACTH) levels were measured and the values were normal. A biopsy was taken from the buccal mucosa with the aim of excluding melanoma. Histopathological examination revealed lentiginous proliferation of melanocytes. Inflammatory changes or malignant features were not noted in any area. A diagnosis of LH syndrome was made based on the clinical presentation of lesions coupled with the absence of systemic findings.

An Er:YAG laser with a wavelength of 2940 nm (Fotona Fidelis Plus III, Slovenia) was used because of its capability of superficially ablating oral soft tissues to treat oral pigmentation, especially on the lips, soft palate, and buccal mucosa, bilaterally. The parameters used were 120 mJ output energy, 10 Hz frequency, 1000 *μ*s pulse duration, and a 0.8 mm spot size with the noncontact hand-piece. The fluence was 25 J/cm^2^ and the lasing was continued until the pigmentation on the intervention area was visibly ablated as there is no accumulation of energy or heat by Er:YAG lasers. The treatment was performed under local anesthesia with no water and air spray. No sutures or other means of bleeding control were needed at the end of the procedure. Postoperative healing was uneventful, only mild “burning” sensation was reported by the patient for a few hours postoperatively. The reason of the need for anesthetics is that the water spray was turned off in order to obtain hemostasis. The procedure was straightforward, scanning all the pigmented areas of the oral cavity and the lips, ablating the superficial layers of the tissues including the buccal mucosa and the soft palate.

**Figure 2:**
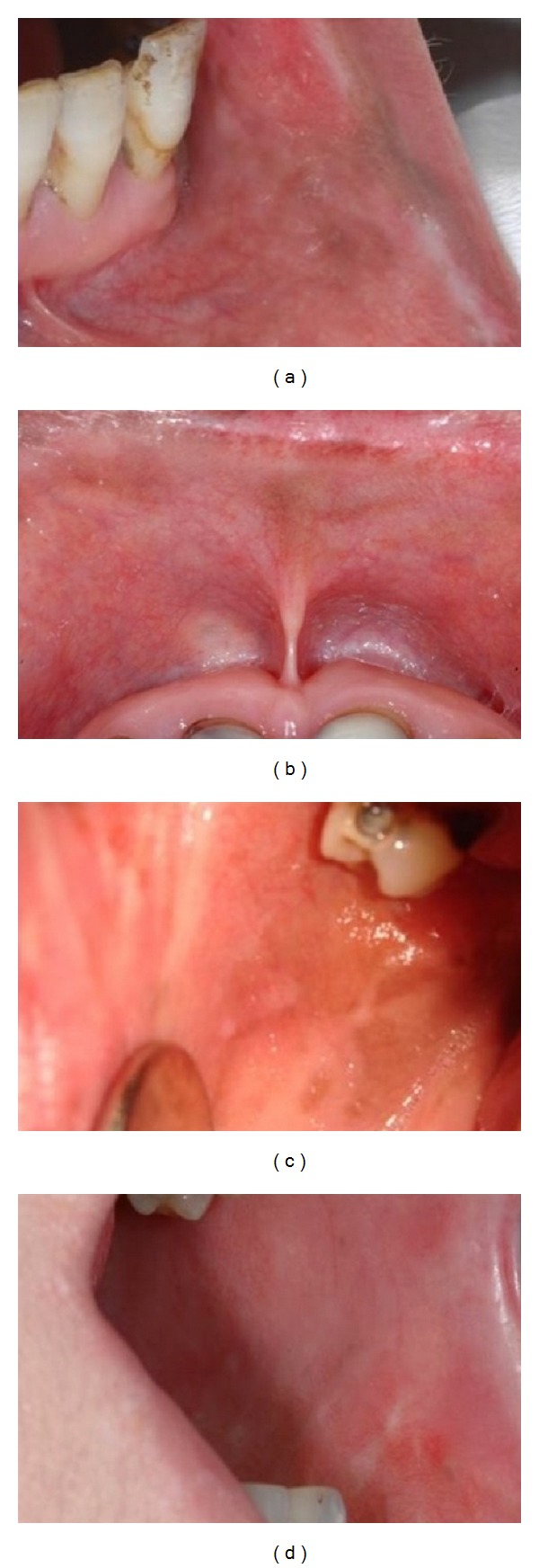
The healing process occurred without any complication and the entire pigmentation had been cleared (14th day postoperatively).

**Figure 3:**
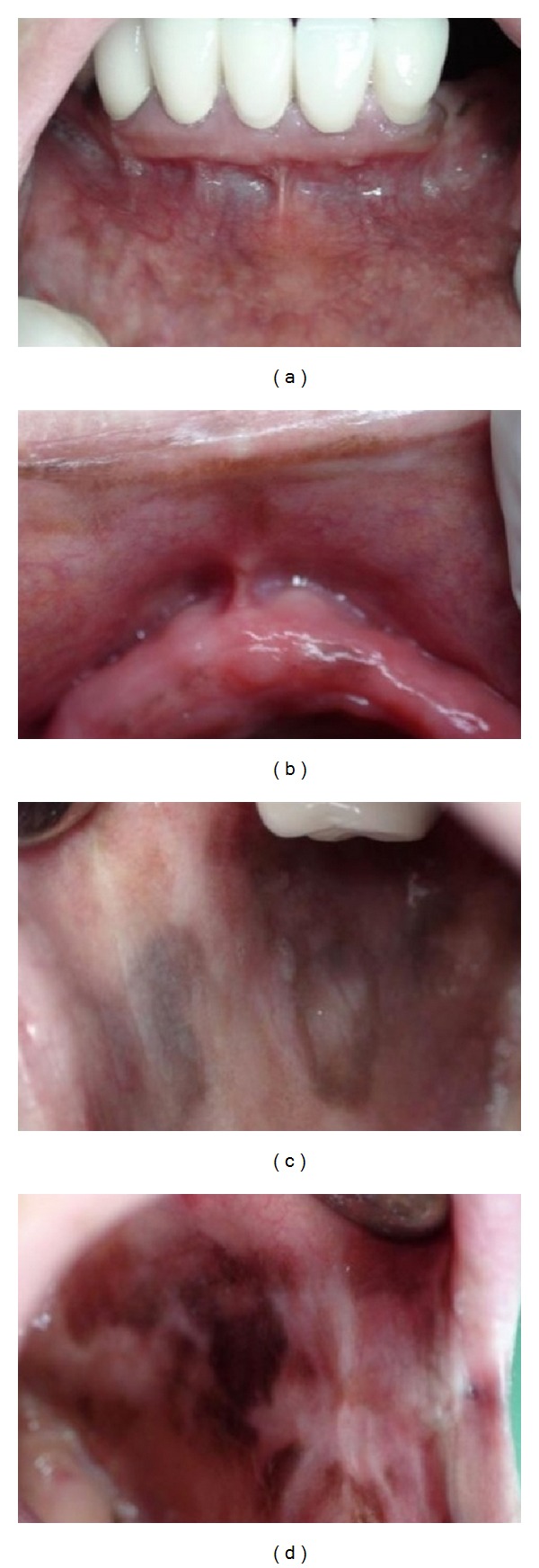
By the 12th month of follow-up, the pigmentation had recurred.

As the area of pigmentation was rather large the surgical treatment was performed in 4 sessions. In each session one quadrant was chosen and cleared in order to reduce scarring. After the 4th session, the healing process occurred without any complication and the entire pigmentation had been cleared (Figure 2). Two months later, however, a small pigmented area was seen, and it was cleared again in one appointment. By the 12th month of followup, the pigmentation had recurred, being observed in an area close to half of the initial situation (Figure 3).

## 3. Discussion

Diagnosis of Laugier-Hunziker syndrome must be established to exclude underlying systemic pathologic conditions such as Addison's disease, Albright's syndrome, and Peutz-Jeghers syndrome [4]. Diffuse oral pigmentation may also be associated with systemic intake of drugs such as tetracyclines, antimalarials, amiodarone, chemotherapeutic agents, oral contraceptives, phenothiazines, azidothymidine, and ketoconazole [5]. A correct diagnosis will resolve the drug induced oral mucosal pigmentation following the suspension of the causative drug [6]. No drugs were being taken by our patient.

Smoking may produce oral pigmentation, although it is usually confined to the anterior attached gingival and not associated with pigmentation in other parts of the body [7]. The patient has been nonsmoker for her entire life.

The majority of pigmentations associated with LHS do not require any treatment, it necessary to reassure the patient of the benign behavior of the lesions. In the present case, the pigmentations were treated by laser because the patient was concerned about her appearance. She was unable to socialize being unhappy with her “unhealthy look”. The treatment was carried out in order to improve patient's quality of life as requested by the patient herself. Unfortunately, this treatment rendered only a transitory clearance of the pigmentations, as the lesions recurred later on, and no other attempt to treat the patient was made.

Treatment using the Q-switched neodymium: yttrium-aluminum-garnet (Nd:YAG) laser [5], Q-switched alexandrite laser (QSAL) [2, 8, 9], and cryosurgery [10] has been reported in only a small number of patients.

Er:YAG laser was the tool of choice as it is very superficially absorbed by the oral tissues, not risking the underlying structures. Even though the procedure required anesthetic solutions to be administered, the thermal effects of Er:YAG are minimal, thus not delaying the healing but maintaining hemostasis during the operation. Other wavelengths like Nd:YAG or diodes which are absorbed in hemoglobin and melanin could also be used in this case, however the thermal side effects are more, which may cause delayed wound healing [11]. This procedure may be very difficult or even not possible to be done by scalpel surgery while with the Er:YAG it is simple for an experienced laser user.

There seems to be no risk of induction of malignancy with Er:YAG laser ablation in this case because the diagnosis of the case was already known to be non-premalignant after the biopsy taken and there is no biomodulation effect of 2940 nm wavelength as it is highly absorbed by water, not letting the energy penetration into the tissues. Another biopsy after the treatment was not justified because of these reasons and was not done.

There are few report of treatment by surgical laser with no recurrence within 12-month follow-up [2, 9]. Further studies are therefore called for with long term follow-up in order to confirm whether the effects of the surgical treatment of pigmentations associated with LHS are permanent or not.

The main limitation of this case report was the lack of the related references. There were a few published LH cases who were treated with other types of lasers than Er:YAG laser whereas there was only one study evaluating the treatment of Laugier-Hunziker Syndrome with the Q-switched alexandrite laser whose study group was consisted of 22 Chinese patients [2]. For further investigations, there is a need for a study with higher study population which will compare the treatment outcomes of different types of lasers.

As recurrences were observed in the present case after 12 months of followup it may be reasonable to consider that this acquired pigmentation is to be persistent for life with a tendency to be refractory to a surgical intervention. It can be concluded that the reason of the recurrence in this case may be the superficial removal of tissue layers with Er:YAG which was thought to be advantageous as it is safe. The advantage of using the Er:YAG laser is being able to see the pigmented areas easily; however, the disadvantage is that it is only possible to see them clinically. The advantage of another laser could be that it could interact with the pigmentation on molecular scales which could prevent recurring. Other surgical tools or another laser with a different choice of wavelength to interact with the deeper pigment content of the lesions could be investigated for a longer term successful result.
